# Ectonucleotidase Modulation of Lymphocyte Function in Gut and Liver

**DOI:** 10.3389/fcell.2020.621760

**Published:** 2021-01-21

**Authors:** Luiz Eduardo Baggio Savio, Simon C. Robson, Maria Serena Longhi

**Affiliations:** ^1^Laboratory of Immunophysiology, Biophysics Institute Carlos Chagas Filho, Federal University of Rio de Janeiro, Rio de Janeiro, Brazil; ^2^Department of Anesthesia, Critical Care & Pain Medicine, Beth Israel Deaconess Medical Center, Harvard Medical School, Boston, MA, United States; ^3^Division of Gastroenterology, Department of Medicine, Beth Israel Deaconess Medical Center, Harvard Medical School, Boston, MA, United States

**Keywords:** ENTPD1, Treg, Th17, Crohn's disease, autoimmune hepatitis

## Abstract

Imbalance between regulatory and effector T lymphocytes contributes to loss of immunotolerance and plays a permissive role in the initiation, perpetuation, and progression of chronic inflammatory diseases and autoimmune disorders. Regulatory/effector cell balance is governed by the CD39 ectonucleotidase, the prototype member of the NTPDase family that hydrolyzes ATP and ADP into AMP, subsequently converted into adenosine by CD73. Generation of adenosine impacts T-cell function as it contributes to the mechanism of suppression of Tregs and confers regulatory properties to pathogenic Th17-cells. CD39 cell distribution, mechanism of regulation and impact on inflammatory and regulatory signaling pathways are also discussed here. Innovative therapeutic strategies to boost CD39 levels and activity by either administering soluble ADPases or interfering with CD39 inhibitory signals are reviewed. Restoration of CD39 levels and function has enormous translational and clinical implications and should be regarded as an additional form of treatment to be deployed in the chronic inflammatory setting. The key role of CD39 in immunoregulation in the context of Crohn's disease, one of the most frequent manifestations of inflammatory bowel disease, and autoimmune hepatitis, an autoimmune disorder of the liver, is reviewed and discussed here.

## Introduction

After release in the extracellular space, adenosine triphosphate (ATP), a mediator of multiple inflammatory processes, is hydrolyzed to adenosine diphosphate (ADP) and adenosine monophosphate (AMP), subsequently converted into immunosuppressive adenosine. Hydrolysis of ATP and other ectonucleotides into AMP and adenosine is catalyzed by ectonucleotidase enzymes that include the ecto-nucleoside-triphosphate diphosphohydrolases (ENTPDases), the ecto-5′-nucleotidase (NT5E)/CD73, the ectonucleotide-pyrophosphate phosphodiesterases, NAD glycohydrolyses, CD38/NADase, alkaline phosphatase, adenylate kinase, the nucleoside diphosphate kinase, and the ecto-F1-F0 ATP synthases (Moser et al., 2001). The ENTPDase family includes eight enzymes: ENTPD1/CD39 that converts ATP and ADP into AMP; ENTPDase 3 and 8, which preferentially catalyze ATP; and ENTPDase 2 that hydrolyzes ATP only (Kukulski et al., 2015); all these are located on the cell surface. ENTPDases 4-6 have intracellular location, whereas ENTPDases 5 and 6 are secreted upon heterologous expression.

This review will focus on ENTPDase1/CD39, the ENTPDase family prototype, which is expressed on the vasculature and on innate and adaptive immune cell subsets. Due to the ectonucleotidase activity that enables hydrolysis of pro-inflammatory ATP to ultimately generate adenosine, CD39 is regarded as a key modulator of the immune system, contributing to the balance between regulatory and effector lymphocytes in physiological conditions. Among immune cells, CD39 is largely expressed on B cells and monocytes/macrophages, followed by CD4 lymphocytes and, to a lesser extent, NK and CD8 cells. We will review and discuss the pivotal role of this ectoenzyme in governing regulatory and effector T cell responses in inflammatory and autoimmune conditions, including Crohn's disease and autoimmune hepatitis (AIH). Signaling pathways involved in CD39 regulation and induction in different immune cell types are also reviewed and discussed.

## Chemical Structure and Immune Cell Distribution

**Figure 1:**
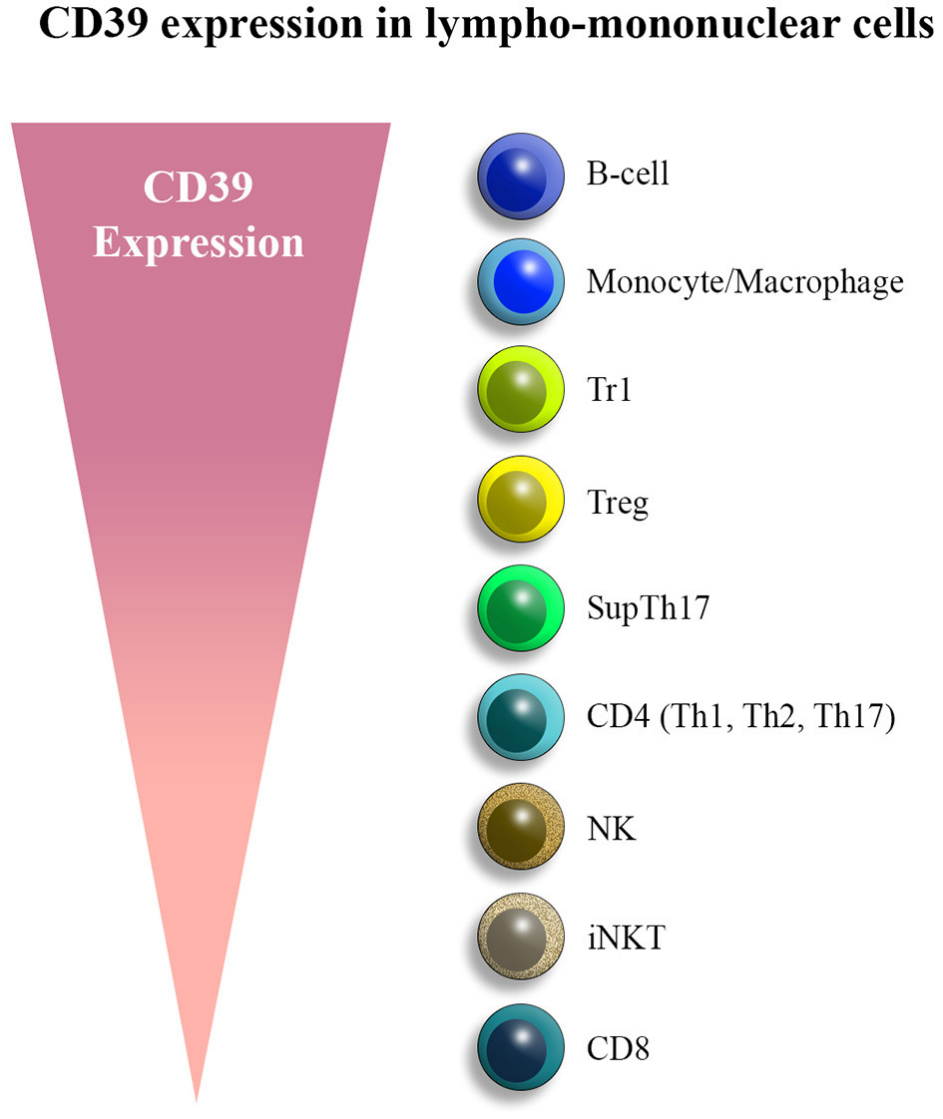
CD39 expression in lympho-mononuclear cells. CD39 is mainly expressed by B cells, monocytes/macrophages, and various T cell subsets, chiefly T regulatory type 1 (Tr1) cells, regulatory T cells (Tregs), and suppressor Th17 (supTh17) cells. Lower levels of expression are noted in NK subsets and CD8 lymphocytes.

CD39 was initially purified as soluble ATP diphosphohydrolase (apyrase) from potato tubers (Handa and Guidotti, 1996); subsequently, ATP diphosphohydrolases were isolated from porcine pancreas and bovine aorta and found sharing sequence homology with CD39 cDNA, obtained from human endothelial cells. CD39 is present in different variants, deriving from differential splicing (Kaczmarek et al., 1996). Akin to other NTPDases, CD39 is composed of five highly conserved sequence domains, which are involved in the formation of the active site and in the catalysis of extracellular nucleotides. CD39 is anchored to the cell membrane through two transmembrane domains, which are essential to maintain the catalytic activity as well as the specificity for the substrate (Grinthal and Guidotti, 2002, 2006). The ectoenzyme undergoes functional modifications, which are key in conferring catalytic activity; these include glycosylation and, in the case of the N-terminal intracytoplasmic domain, palmitoylation that enables association with the lipid rafts (Kittel et al., 1999; Koziak et al., 2000; Papanikolaou et al., 2005). We have recently shown that CD39 co-localizes with lipid raft markers like caveolin 1 and flotillin 2 in LPS-primed macrophages exposed to ATP (Savio et al., 2020). CD39 is constitutively expressed by various cells of the immune system, chiefly mature B cells (Zacca et al., 2020), monocytes/macrophages, as well as in subsets of CD4 (Clayton et al., 2011), NK cells, and CD8 lymphocytes (Clayton et al., 2011) (Figure 1). CD39 is also present on epidermal dendritic cells, where it protects against skin inflammation (Mizumoto et al., 2002). CD39 expression is enhanced by various factors, as reviewed and discussed in the next section.

Levels of CD39 expression and activity in regulatory and effector cell subsets govern immunohomeostasis; alterations of CD39 might therefore impact Treg and effector cell functionality and, ultimately, disease outcomes like in Crohn's disease and AIH. CD39 is expressed by subsets of CD4 cells, including memory lymphocytes (Zhou et al., 2009) that display a Th1/Th2 and Th17 immunophenotype (Zhou et al., 2009). These findings obtained in the murine setting were also echoed by investigations in human samples, where CD39 was found in CD4 cells with effector memory phenotype (Dwyer et al., 2010). CD39 expression increases with age in CD4 cells that undergo metabolic stress and apoptosis (Fang et al., 2016).

Th17 cells, another effector subset involved in Crohn's disease and AIH liver damage, can also express CD39. There is evidence by others and us that CD39^+^ Th17 cells represent a subpopulation of effector lymphocytes endowed with regulatory/immunosuppressive properties (Chalmin et al., 2012; Longhi et al., 2014). These “suppressor” Th17 cells (supTh17) express both CD39 and CD73 and suppress CD4 T cell effector function through the generation of adenosine. However, due to decreased A_2A_ receptor levels, supTh17 are less responsive to autoregulation mediated by adenosine (Longhi et al., 2014). Generation of Th17 cells in the presence of interleukin (IL)-6 and transforming growth factor beta (TGF-β) results in CD39 upregulation, at variance with differentiation obtained in the presence of IL-6, IL-1β, and IL-23 that is not associated with CD39 upregulation (Chalmin et al., 2012). STAT-3, a transcription factor that regulates Th17 cell differentiation, activates a negative feedback loop that limits the formation of Th17 cells by upregulating CD39 (Purvis et al., 2014). Expression of CD39 by tumor-infiltrating Th17 cells is accompanied by concomitant expression of both RORγt and Foxp3, secretion of Th17-related cytokines, and ability to suppress CD4 and CD8 T cell activation. Tumor infiltration of these cells is related to poor clinical outcome in the context of breast cancer (Thibaudin et al., 2016). In contrast with these findings in the tumor setting, the presence of CD39 in Th17 cells modulates inflammation in human visceral adipose tissue in obese patients (Pandolfi et al., 2016). A role for CD39 in limiting Th17 cell effector potential has been corroborated by the findings of Morianos et al., who reported that both CD39 and CD73 are required for activin-A-induced suppression of pathogenic Th17 cells in experimental autoimmune encephalomyelitis (Morianos et al., 2020). Mechanistically, activin-A regulates hypoxia-inducible factor-1alpha (HIF-1α), which is linked with Th17 cell pro-inflammatory properties (Morianos et al., 2020).

In early studies, expression of CD39 by CD8 cells identified a subset of cytotoxic cells that mediate allo-cytotoxic T lymphocyte or NK-like reactivity (Gouttefangeas et al., 1992). In subsequent investigations, CD39 was reported marking CD8 T cell exhaustion, as shown in a mouse model of chronic lymphocytic choriomeningitis virus infection (Gupta et al., 2015). These findings were echoed by another study from Canale and colleagues, indicating that high levels of CD39 in tumor-infiltrating CD8 cells were associated with features of exhaustion and inhibition of IFN-γ production by responder cells (Canale et al., 2018). Interestingly, in colorectal and lung cancers, the absence of CD39 on CD8 T lymphocytes defines a population that lacks features of chronic antigen stimulation at the tumor site, these cells being regarded as bystanders (Simoni et al., 2018). In a recent study, deficiency of Ffar 2 favored tumorigenesis by impacting gut barrier integrity, and this was associated with the presence of phenotypically exhausted CD8 T cells that expressed CD39 and other co-inhibitory molecules (Lavoie et al., 2020). In contrast, CD103^+^CD39^+^ tumor-infiltrating CD8 cells display a distinct TCR repertoire and kill tumor cells in a MHC-class-I-dependent manner *via* IFN-γ secretion, therefore playing an active role in the control of tumor growth (Duhen et al., 2018). Whether in this setting adenosine enhances the inflammatory response, as previously reported (Ouyang et al., 2013), remains unclear. CD39 is also upregulated on CD8 T cells during induction of peripheral tolerance *in vivo*, this being associated with the release of IL-12 and IL-10 (Noble et al., 2016). In a model of sclerosing cholangitis and biliary fibrosis, genetic deletion of *Cd39* exacerbates liver injury, fibrosis, and ductular reaction in multidrug-resistant-protein-2 (*Mdr2*)^−/−^ mice and is associated with an increase in hepatic CD8 T cells; this phenotype can be reproduced upon administration of αβATP into CD39-sufficient *Mdr2*^−/−^ mice, further supporting the immunomodulatory role of CD39 in cytotoxic CD8 cells (Peng et al., 2017).

In several studies, presence of CD39 in murine and human Tregs has been linked with the suppressive function of these cells (Borsellino et al., 2007; Deaglio et al., 2007; Mandapathil et al., 2010). In humans, CD4^+^CD25^high^CD39^+^ and CD4^+^CD25^high^CD39^−^ cells can both suppress IFN-γ production by responders, whereas only CD4^+^CD25^high^CD39^+^ cells control IL-17 production by effector CD4. These cells are impaired in the peripheral blood of patients with multiple sclerosis (Fletcher et al., 2009) and pustular psoriasis (Han et al., 2018). CD39^+^ Tregs can limit CD62L shedding in T cells that, consequently, remain in the lymph nodes; this phenomenon results in defective sensitization in contact hypersensitivity reactions (Mahnke et al., 2017). In the setting of hepatic metastatic cancer, developed upon portal vein infusion of luciferase-expressing melanoma B16/F10 and MCA38 colon cancer cells, expression of CD39 on Tregs inhibits NK cell activity, therefore playing a permissive role in metastasis growth (Sun et al., 2010). In the autoimmune setting, products from commensal bacteria can modulate the migration of Tregs to the central nervous system *via* CD39 regulation, further corroborating the immunomodulatory role of this ectonucleotidase in the control of inflammatory processes (Wang et al., 2014). In sepsis-associated encephalopathy, CD39 limits excessive cytokine production in the brain (Savio et al., 2017a). Importantly, Tregs obtained from rheumatoid arthritis patients unresponsive to methotrexate express low levels of CD39 and, consequently, generate less adenosine and display reduced suppressor ability compared with Tregs isolated from methotrexate responders (Peres et al., 2015); this suggests a link between methotrexate unresponsiveness and low CD39 levels. This CD39 defect results from impairment of TGF-β signaling (Peres et al., 2018) due to reduced TGF-βR2 and CREB1 and decreased SMAD2 and CREB phosphorylation in Tregs. The role of TGF-β in CD39 upregulation by Tregs was found to be counteracted by reactive oxygen species (ROS)-driven autophagy (Gerner et al., 2020).

CD39 is also expressed in T regulatory type 1 (Tr1) cells, a regulatory subset predominant during chronic inflammation and recovery (Roncarolo et al., 2006). CD39 promotes Tr1 cell differentiation and contributes to the suppressor ability of these cells through the generation of adenosine (Mascanfroni et al., 2015). Activated γδ T cells are known to protect from autoimmunity, this property being linked to CD39 expression (Ujiie and Shevach, 2016).

CD39 can also abrogate γδ TCR agonistic activity of both self and microbial phosphoantigens, revealing a novel regulatory function of the CD39 ectoenzyme (Gruenbacher et al., 2016).

In contrast, when considering NK cells, a subset-mediating hepatic ischemia reperfusion injury, deficiency of CD39 results in abrogation of IFN-γ secretion by these cells with consequent limitation of tissue damage (Beldi et al., 2010). Notably, CD39^+^ NKT cells mediate hypoxic lung injury *via* IFN-γ and IL-17 secretion and damage is not observed in *Cd39*^−/−^ mice as well as in NKT cell-immunodeficient mice. Similar findings are noted upon adoptive transfer of *Cd39*^−/−^ iNKT cells (Nowak-Machen et al., 2013).

## Regulation of CD39

CD39 can be regulated by various mechanisms that operate at the genetic, transcriptional, and post-transcriptional level.

Presence of a single nucleotide polymorphism (SNP) tagging low *CD39* mRNA levels has been associated with predisposition to Crohn's disease (Friedman et al., 2009). Subsequent studies have shown that highly heritable cell populations express CD39 (Orru et al., 2013), being CD39 levels under genetic control in regulatory lymphocytes (Roederer et al., 2015). The evidence that CD39^+^ Tregs are present in naïve compartments such as cord blood and thymus and that the frequency of CD39^+^ Tregs is stable over time further strengthens the postulate that CD39 is regulated at the genetic level. In this regard, Tregs from donors with a GG genotype, corresponding to high CD39 levels, are more effective at controlling IFN-γ and IL-17 production by effector cells, when compared with Tregs derived from donors with AA genotype (tagging low CD39 levels) (Rissiek et al., 2015).

**Figure 2:**
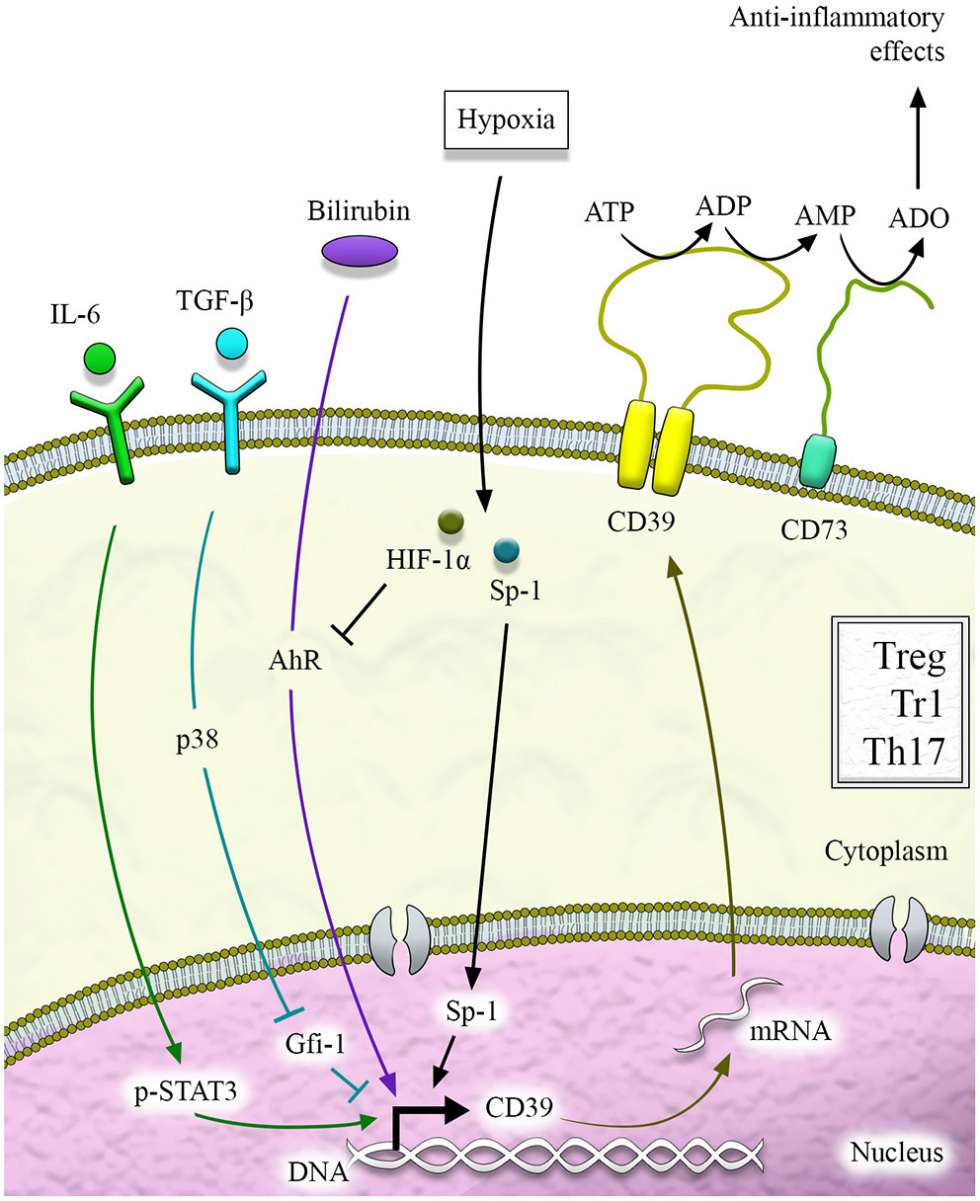
CD39 regulation. CD39 regulation at the transcriptional level is mediated by different pathways that include aryl hydrocarbon receptor (AhR) signaling activation and cytokines like IL-6, which activates STAT-3, and TGF-β, which downregulates Gfi-1. STAT-3 and Gfi-1 are transcription factors with opposing effects on CD39 induction. Hypoxia-inducible factor-1alpha (HIF-1α) and hypoxia play a dual role, sustaining CD39 induction *via* specificity protein-1 (Sp1) or limiting it upon inhibition of AhR signaling.

Several mechanisms can regulate CD39 at the transcriptional level, and these include activation of aryl hydrocarbon receptor (AhR), a modulator of toxin responses and adaptive immunity (Figure 2). Activation of AhR in the presence of TGF-β1 can induce Tregs that suppress through CD39 (Gandhi et al., 2010). The importance of AhR activation for CD39 upregulation has also been provided by the evidence of increased CD39 levels in Th17 cells exposed to unconjugated bilirubin (UCB), an immunometabolite with antioxidant and regulatory properties that also serves as AhR endogenous ligand (Longhi et al., 2017). Inhibition of AhR after exposure to CH223191 (an AhR antagonist) partially limits UCB-induced CD39 upregulation (Longhi et al., 2017). AhR responsive elements have also been found in the CD39 promoter of Tr1-cells that express CD39 in an AhR-dependent manner.

Additional control over CD39 is operated by hypoxia (Figure 2), as confirmed by the identification of a region of the CD39 promoter that is key to induction by hypoxia (Eltzschig et al., 2009). In this regard, the specificity protein-1 (Sp1) has been reported as the transcription factor involved in hypoxia-induced CD39 upregulation (Eltzschig et al., 2009). Evidence of Sp1-mediated CD39 upregulation has been provided in models of cardiac ischemia (Eltzschig et al., 2009) and hepatic ischemic preconditioning (Hart et al., 2010). Molecular blockade of Sp1 *via* siRNA resulted in reduced CD39 induction and increased hepatic injury *in vivo* (Hart et al., 2010). Additional investigations in the context of chronic inflammation like inflammatory bowel disease (IBD) have shown that hypoxia and HIF-1α impair UCB-induced CD39 upregulation in Th17 cells that become less responsive to AhR activation (Xie et al., 2018).

CD39 induction has also been linked to exposure to statins in endothelial cells (Kaneider et al., 2002) and to transcription factors like STAT-3 and Gfi-1 that respectively support and repress CD39 expression in Th17 cells (Chalmin et al., 2012) (Figure 2). Additional regulation of CD39 derives upon binding of the ectonucleotidase N-terminus with Ran-binding protein M (RanBPM) (Wu et al., 2006). As a result of this binding, recombinant CD39 ENTPDase activity is substantially diminished (Wu et al., 2006). Control over CD39 expression is also provided by cytokines like IL-27 (Mascanfroni et al., 2015; Park et al., 2019), TGF-β, IL-6 (Thibaudin et al., 2016), and IL-35 (Kochetkova et al., 2010) and by phosphoantigens (Gruenbacher et al., 2016). There is mounting evidence that CD39 is also regulated at the post-transcriptional level upon inhibition of phosphodiesterases (Baek et al., 2013) as well as by an antisense to LMP1, one of the Epstein-Barr virus latent genes (Masciarelli et al., 2002). Further work is warranted to clearly define the role of post-transcriptional regulation in the control of CD39 levels and ectoenzymatic activity.

Table 1 summarizes CD39 inducers and suppressors in various immune and endothelial cells.

**Table 1:** CD39 inducers and suppressors in immune and endothelial cells.

**Cell Type**	**CD39 Inducers**	**CD39 Suppressors**
Th1	Ryegrass pollen (Mittag et al., 2010); anti-CD3/CD28, ITE (Goettel et al., 2016)	Unclear
Th2	Ryegrass pollen (Mittag et al., 2010)	Unclear
Th17	UCB (Longhi et al., 2017); IL-6/STAT-3 (Chalmin et al., 2012); TGF-β (Chalmin et al., 2012)	Gfi-1, IL-1β, IL-23 (Chalmin et al., 2012); HIF-1α (Xie et al., 2018)
CD8^+^	IL-6+IL-27 (Canale et al., 2018); TGF-β (Duhen et al., 2018); anti-CD3/CD28 *via* JNK and NFkB signaling (Bai et al., 2015)	Unclear
Treg	FOXP3; TGF-β; IL-27 (Park et al., 2019); IL-35 (Kochetkova et al., 2010); AhR (Gandhi et al., 2010), IL-33 (Biton et al., 2016)	ROS (Gerner et al., 2020)
Tr1	IL-27 (Mascanfroni et al., 2015); AhR (Mascanfroni et al., 2015)	Hypoxia/HIF-1α (Mascanfroni et al., 2015)
B	RanBPM (Wu et al., 2006); LMP1(Masciarelli et al., 2002)	Unclear
NK	IL-6 (Zheng et al., 2020)	Unclear
iNKT	Anti-CD3, IL-12, IL-18, A_2A_ agonists (Yu et al., 2018)	Unclear
Macrophage	STAT-3 and NF-κB (Savio et al., 2017b); AhR (Takenaka et al., 2019)	PDE3 (Baek et al., 2013)
DC	IL-27 *via* STAT-3 (Mascanfroni et al., 2013), TGF-β (Torres-Aguilar et al., 2010), IL-27 (Alameddine et al., 2019)	IL-10 (Torres-Aguilar et al., 2010)
Neutrophil	IL-6 (Lazar et al., 2016)	Unclear
Endothelial	Statins (Kaneider et al., 2002); Sp1 (Eltzschig et al., 2009)	Thrombin (Kaneider et al., 2002); IL-6; TGF-β (Roy et al., 2018); phenolic compounds (Saji et al., 2019)

*Th1, T helper type 1; Th2, T helper type 2; Th17, T helper type 17; UCB, unconjugated bilirubin; FOXP3, forkhead box P3; Treg, T regulatory cell; Tr1, T regulatory type 1; AhR, aryl hydrocarbon receptor; HIF-1α, hypoxia inducible factor 1alpha; RanBPM, Ran-binding protein M; LMP1, latent membrane protein 1; NK, natural killer; iNKT, invariant natural killer T; NF-κB, nuclear factor kappa-light-chain enhancer of activated B cells; PDE3, phosphodiesterase 3; DC, dendritic cell*.

## Crohn's Disease

IBD is a chronic inflammatory condition that results from altered interactions between the gut microbiota and the immune system in genetically predisposed individuals (Cho, 2008a,b). Crohn's disease, one of the major IBD forms, presents with a relapsing-remitting course, which is associated with considerable morbidity, increased cancer risk, and death. Imbalance between Th17 cells and Tregs has been extensively documented (Maul et al., 2005; Eastaff-Leung et al., 2010) and thought to contribute to disease pathogenesis. A wealth of studies conducted over the years has supported the role of CD39 in governing this balance in both experimental colitis models and Crohn's disease in humans.

In the context of experimental colitis chemically induced by dextran sulfate sodium (DSS), *Cd39*^−/−^ mice undergo a more severe course of the disease, which is reverted, at least in part, upon administration of apyrase with ectoenzymatic activity comparable with CD39 (Friedman et al., 2009). In a subsequent study, we showed that UCB has beneficial effects in the recovery phase of the disease, these depending on AhR and being mediated by CD39 (Longhi et al., 2017). Furthermore, human CD39 overexpression under the control of the H-2k^b^ promoter results in a less-severe course of DSS colitis in mice and protects from hypoxia-induced tissue damage (Robles et al., 2020). In experimental colitis induced by trinitrobenzene-sulfonic-acid (TNBS), a chemical with haptenic properties, *NOD/scid/gamma* mice transgenic for human HLA-DR2, reconstituted with HLA-DR2^+^ CD4 cells and administered 2-(1′H-indole-3′-carbonyl)-thiazole-4-carboxylic acid methyl ester, an AhR endogenous ligand, undergo a more benign course of the disease, when compared with controls (Goettel et al., 2016). These beneficial effects are mediated by CD39 (Goettel et al., 2016). However, *Cd39* global deficiency in C57BL6 mice resulted in protection from colitis, suggesting that differences in genetic background may account for different impacts on CD39 and disease outcome (Kunzli et al., 2011). In experimental colitis induced by adoptive transfer of CD45RB^high^ cells, administration of *Cd39*^−/−^ Tregs resulted in less-effective control of the disease, when compared with WT Tregs (Gibson et al., 2015). In contrast, administration of Tregs obtained from human CD39 transgenic mice led to disease amelioration, as reflected by reduced disease activity index and histology score (Robles et al., 2020).

Enrichment in the low CD39-expressing AA genotype was observed in Crohn's disease patients and found to be associated with disease predisposition (Friedman et al., 2009).

Decrease in the proportion of supTh17 (i.e., CD39^+^ Th17 cells) has been reported by us in the circulation and lamina propria of Crohn's disease patients (Longhi et al., 2014). Furthermore, in Crohn's disease, Th17 cells display defective response to AhR activation *via* UCB, this being reflected by inadequate upregulation of CD39 (Longhi et al., 2017). The reason for this poor responsiveness relies, at least to some extent, on aberrantly high levels of inflammation-induced HIF-1α that induces drug transporters like multidrug resistance 1 (MDR1) and multidrug resistance protein 4 (MRP4) in Th17 cells derived from Crohn's disease patients. MDR1 and MRP4 favor the efflux of immunometabolites like UCB from cells, this resulting in impaired CD39 upregulation (Xie et al., 2018). Exposure of cells to HIF-1α molecular blockade with siRNA reverts this phenomenon, also under hypoxic conditions (Xie et al., 2018). Low levels of CD39 expression are reported in Tregs derived from Crohn's disease patients with active disease; in contrast, Tregs obtained from treatment responders express CD39 at higher levels (Gibson et al., 2015). A decrease in CD39 expressing cells was recently reported in a cohort of pediatric IBD patients along with other immunological alterations like infiltration of phosphodiesterase 4B and TNF-α expressing macrophages, impaired cyclic-AMP response signaling, and platelet aggregation (Huang et al., 2019).

Based on these studies, immunotherapeutic strategies could be applied to restore CD39 levels and activity. As high levels of HIF-1α can induce upregulation of drug transporters like MDR1 and MRP4 that favor exit of immunometabolites out of cells (Xie et al., 2018), pharmacological approaches aimed at inhibiting these drug transporters might be considered. In this regard, we tested the effects of ritonavir, a nonspecific MDR1 and MRP4 inhibitor, on Th17 cell ability to respond to AhR activation. We found that ritonavir restores Th17 cell response to UCB by upregulating CD39, even under hypoxic conditions (Xie et al., 2018). Another potentially effective approach would entail direct boosting of CD39 activity by administration of soluble apyrase that prevents DSS colitis in *Cd39*^−/−^ mice (Friedman et al., 2009). Recently, we have reported that administration to DSS mice of APT102—the extracellular domain with improved ADPase activity of human nucleoside triphosphate diphosphohydrolase 3, a member of the CD39 family—augments the beneficial properties of UCB (Robles et al., 2020), while boosting the proportion of CD4^+^FOXP3^+^, CD4^+^CD39^+^, and CD4^+^LAG3^+^ cells in the lamina propria (Robles et al., 2020). *In vitro* exposure of Tregs and Th17 cells to APT102 boosts the effects of AhR stimulation in Crohn's disease patients' samples, as reflected by increased CD39, FOXP3, and LAG-3 levels in these cells (Robles et al., 2020).

Expression of CD39 in CD161^+^CD4^+^ cells identifies effector Th17 cells, the frequency of which is elevated in the peripheral blood and lamina propria of Crohn's disease patients (Bai et al., 2014). Notably, exposure to anti-CD3/CD28 induces CD39 in CD8 cells, another effector subset involved in Crohn's disease immunopathogenesis (Bai et al., 2015). These cells inhibit production of IFN-γ by CD39^−^CD8^+^ *via* A_2A_ receptor activation (Bai et al., 2015).

## Autoimmune Hepatitis

AIH is an organ-specific autoimmune disorder characterized by hypergammaglobulinemia, positivity for autoantibodies, and histological presence of interface hepatitis (Heneghan et al., 2013). AIH follows a relapsing-remitting course and, when untreated, may lead to severe organ damage resulting in end-stage liver disease and transplantation. Treg impairment in AIH permits cytotoxic CD8 and CD4 effector cells, including the Th17 subset, to perpetrate liver damage (Ma et al., 2006; Longhi et al., 2007; Zhao et al., 2011). Defects in CD39 expression and activity underlie this immune dysregulation. In previous studies, we showed numerical and functional impairment of CD39^+^ Tregs that fail to generate adenosine, do not control Th17 cell immunity and are more prone to acquire phenotypic features of effector cells when exposed to a pro-inflammatory challenge (Grant et al., 2014). Akin to Tregs, effector Th17 cells, one of the effector subsets involved in AIH liver damage, display low levels of CD39, this being associated with impaired adenosine generation and ability to acquire regulatory properties (Liberal et al., 2016). We have recently shown that defective CD39 levels in AIH Tregs and Th17 cells derive from altered AhR signaling (Vuerich et al., 2020). These alterations result from an aberrant increase in aryl hydrocarbon receptor repressor in Tregs and HIF-1α in Th17 cells. Both factors inhibit AhR. Furthermore, in AIH Tregs, there is a marked increase in estrogen receptor alpha (Erα), one of AhR alternative binding partners. Preferential binding of AhR to Erα rather than aryl hydrocarbon receptor nuclear translocator, the classical AhR binding partner, leads to impaired CD39 upregulation (Vuerich et al., 2020); this possibly accounting for low CD39 levels in Tregs.

## Concluding Remarks

Important basic and translational studies support the chief role of CD39 in governing the balance between effector and regulatory cells. While normal or heightened CD39 levels and activity result in beneficial immunological effects, impaired CD39 expression and function likely result in effector/regulatory cell imbalance that contributes to perpetuation and progression of tissue damage. Restoring CD39 levels and activity by administering exogenous ADPases or interfering with pathways that ultimately inhibit CD39 (i.e., hypoxia/HIF-1α) or do not favor its full upregulation (i.e., increase in Erα levels) represent innovative strategies to treat chronic inflammatory and autoimmune conditions like Crohn's disease and AIH. Further studies are warranted to dissect the mechanisms leading to these alterations and identify innovative and more effective approaches to correct these and halt inflammatory/autoimmune damage.

## Author Contributions

LS drafted the manuscript. SR and ML reviewed and edited the manuscript. All authors contributed to the article and approved the submitted version.

## Conflict of Interest

The authors declare that the research was conducted in the absence of any commercial or financial relationships that could be construed as a potential conflict of interest.
